# The Vertebral Column, Ribs, and Sternum of the African Giant Rat (*Cricetomys gambianus* Waterhouse)

**DOI:** 10.1155/2013/973537

**Published:** 2013-10-28

**Authors:** Matthew Ayokunle Olude, Oluwaseun Ahmed Mustapha, Temitope Kehinde Ogunbunmi, James Olukayode Olopade

**Affiliations:** ^1^Department of Veterinary Anatomy, College of Veterinary Medicine, Federal University of Agriculture, Abeokuta 110124, Ogun, Nigeria; ^2^Department of Veterinary Anatomy, Faculty of Veterinary Medicine, University of Ibadan, Ibadan 200213, Oyo, Nigeria; ^3^Marine Biological Laboratory, Woods Hole, MA, USA

## Abstract

Examined bones were obtained from eight adult African giant rats, *Cricetomys gambianus* Waterhouse. Animals used had an average body mass of 730.00 ± 41.91 gm and body length of 67.20 ± 0.05 cm. The vertebral formula was found to be C_7_, T_13_, L_6_, S_4_, Ca_31–36_. The lowest and highest points of the cervicothoracic curvature were at C_5_ and T_2_, respectively. The spinous process of the axis was the largest in the cervical group while others were sharp and pointed. The greatest diameter of the vertebral canal was at the atlas (0.8 cm) and the lowest at the caudal sacral bones (2 mm). The diameter of the vertebral foramen was the largest at C_1_ and the smallest at the S_4_; the foramina were negligibly indistinct caudal to the sacral vertebrae. There were 13 pairs of ribs. The first seven pairs were sternal, and six pairs were asternal of which the last 2-3 pairs were floating ribs. The sternum was composed of deltoid-shaped manubrium sterni, four sternebrae, and a slender processus xiphoideus. No sex-related differences were observed. The vertebral column is adapted for strong muscular attachment and actions helping the rodent suited for speed, agility, dexterity, and strength which might enable it to overpower prey and escape predation.

## 1. Introduction

The African giant rats (AGR) are in the murid group of the order Rodentia, characterized by remarkable features such as their vision in the dark, keen sense of olfaction, sheer size, and social acceptability which has increased the drive for basic research data in the past decade. 

The AGR which is an omnivorous nocturnal rodent has been marked as Africa's second most hunted microlivestock [1] and have evolved many locomotive features adapted for its survival [1, 2]; it is observed to easily curl up to bite or escape, jump, and climb high walls and great agility (personal observation). The skeletal system provides the rigid framework of interconnected bones and cartilage that protects and supports the internal organs and provides attachment for muscles and, hence, plays a major part in this rodent's locomotion and ultimate survival.

There are many macroanatomical investigations on the skeletal system of mammals, including the rabbit, the guinea pig, the mink [3], the badger [4, 5], the porcupine [6], the hedgehog [7], and the mole rat [8], mostly bordering on skull studies. The literature on the macroanatomical features of the skeletal system in African giant rat exists with work on some aspects of the osteometry and morphology of the neurocranium [9], pelvic limb [10], and forelimbs [11], but features of the vertebrae, ribs, and sternum remain unstudied in the African giant rats. The aim of this present study, therefore, is to investigate the skeletal anatomy of the axial skeleton in the African giant rats thereby advancing the osteological studies of this rodent and to contribute to the information in this field.

## 2. Materials and Methods

Examined bones were obtained from eight adult African giant rats without regard to sex. The animals were not deliberately deprived of life, but rather, bones were obtained after other scientific experiments, and so it was not necessary to acquire permission from the Bioethics Committee. Maceration of bones was carried out by a modified method of Onar et al. [12, 13] and Olopade and Onwuka [14] to remove muscles, ligaments, and tissues. Anatomical descriptions were adapted to the method of Özkan [8], Onar et al. [13], Olopade and Onwuka [14], Hebel and Stromberg [15], Endo et al. [16], Fernandes et al. [17], and Wysocki [18] with the aid of metric instruments. 

## 3. Results

Animals used had an average body mass of 730.00 ± 41.91 gm and body length of 67.20 ± 0.05 cm. The vertebral formula was found to be C_7_, T_13_, L_6_, S_4_ and variable number of coccygeal bones Ca_31–36_. The lowest point of the cervicothoracic curvature was at C_5_, while the highest point of the spine was at the T_2_ while in the natural position; the highest position was at thoracolumbar junction. The spinous process of the axis was the largest in the cervical group; others were sharp and pointed. There were no sex-related differences observed in this study.

### 3.1. Cervical Vertebrae

There were 7 cervical vertebrae; the atlas and the axis were the largest while the rests were shorter and wider. The greatest diameter of the vertebral canal was observed at the atlas bone (C_1_) (0.8 cm). 

**Figure 1:**
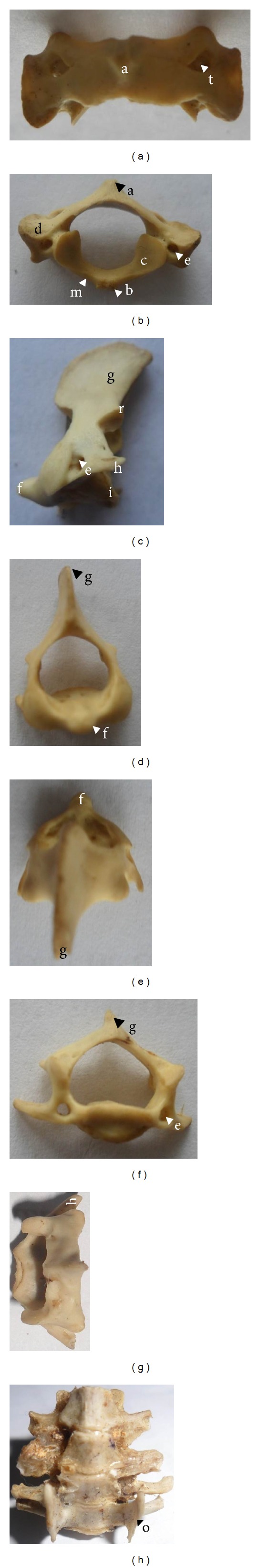
(a) and (b) dorsal and cranial views of the atlas bone, respectively; (c), (d), and (e) lateral, cranial and dorsal views of the axis bone, respectively; (f) and (g): dorsal and cranial views of the typical cervical vertebrae, respectively; (h) ventral view of C4–6 cervical vertebrae. a: dorsal tubercle, b: ventral tubercle, c: cranial articular surface, d: wing of atlas, e: transverse foramen, f: odontoid process, g: spinous process, h: transverse process, i: vertebral body, m: ventral arch, o: cervical rib, r: caudal articular process, and t: alar foramen.

The transverse processes were positioned caudolaterally and penetrated by the transverse canal (Figures 1(b), 1(c), and 1(f)). Homologue of the cervical ribs was observed as caudoventrally directed bony plates from the base of C_6_ (Figure 1(h)). Transverse foramen and alar foramen were present in the atlas. In the axis, the caudodorsally directed spinous process of axis was thicker and higher than the spinous processes of the other cervical vertebrae, and there was a deep groove on the caudal face of the spinous process. There were ventral tubercles on the ventral surfaces of the bodies of the cervical vertebrae (Figure 1(b)). The spinous processes of the axis (C_2_) and the 3rd and 4th cervical vertebrae were slightly caudodorsally directed; the 6th and 7th were slightly craniodorsally directed, while the 5th cervical vertebra was dorsally directed. The small transverse processes of the 2nd–5th cervical vertebrae were caudolaterally directed, and the 6th and 7th cervical vertebrae were laterally directed. The caudal end of the costal process of the transverse process of the 6th cervical vertebra was pointed. Fovea costalis caudalis was present on the 7th cervical vertebra.

### 3.2. Thoracic Vertebrae

**Figure 2:**
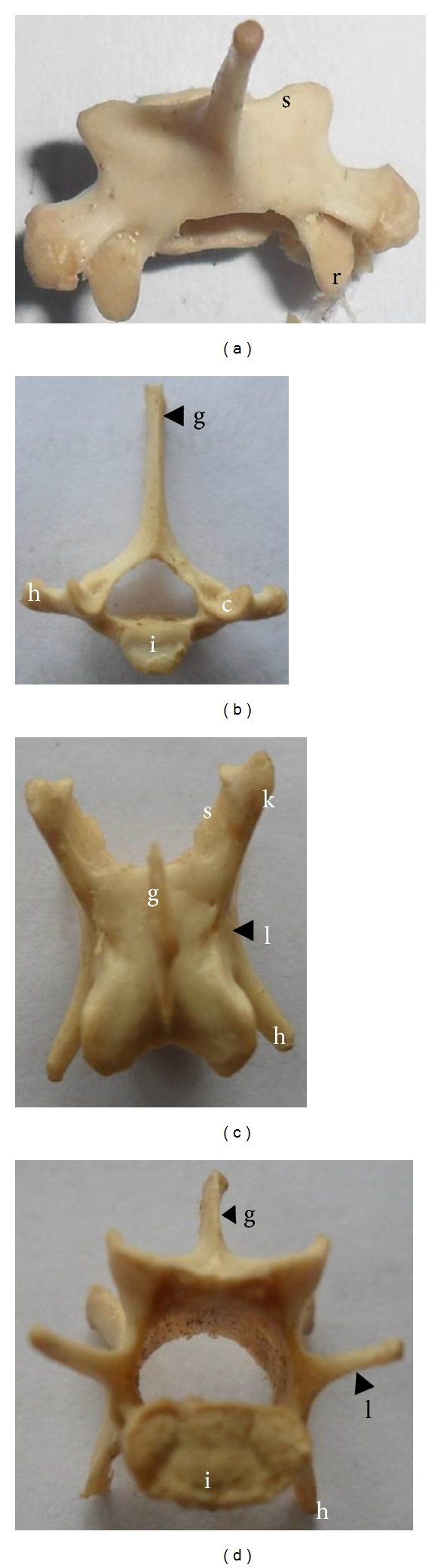
(a) and (b) oblique dorsal and cranial views of the thoracic vertebrae, respectively; (c) and (d) dorsal and cranial views of the lumbar vertebrae, respectively. c: cranial articular surface, g: spinous process, h: transverse process, i: vertebral body, k: mammillary process, l: accessory process, r: caudal articular process, and s: cranial articular process.

There were 13 thoracic vertebrae with a total thoracic column length of 7.4 cm ranging from 0.5 cm to 0.8 cm and the vertebral canal diameter varying between 0.4 and 0.6 cm (Table 1). The spinous process of T_1_ was comparable to the rest of the cervical bones but, from T_2_, it arose to an average of 1.1-1.2 cm bearing a midsagittal notch at its rostral tip and was about 0.3 cm wide (Figures 2(a) and 2(b)). Many of the caudal thoracic bones T_10–13_ distinctly bore mammillary processes arising laterally to the cranial articular process. The caudal ends of the transverse processes were craniolaterally directed. Fovea costalis caudalis was more distinct than fovea costalis cranialis. Spinous processes of the thoracic vertebrae were caudodorsally inclined, and the dorsal processes of the last two thoracic vertebrae were wide and dorsally projected.

### 3.3. Lumbar Vertebrae

There were 6 well-developed lumbar vertebrae with a total average length of 6.1 cm and each vertebra appeared uniform in length. Their spinous process and transverse processes increased in size caudally as the spinous process became more erect. There was observed presence of the mammillary processes on all lumbar vertebrae (Figure 2(c)). The diameter of the vertebral canal had an average range of 0.4–0.6 cm. The level of the spinous processes was the same on all lumbar vertebrae, but their lengths varied between 0.8 and 1.2 cm. The ventral crest was present on all of the lumbar vertebrae. The transverse processes of the 3rd–5th lumbar vertebrae were larger than the other lumbar vertebrae. The transverse process of the last lumbar vertebra was craniolaterally directed. The lumbar vertebrae were slightly larger than the thoracic vertebrae from the dorsal view.

### 3.4. Sacral Vertebrae

**Figure 3:**
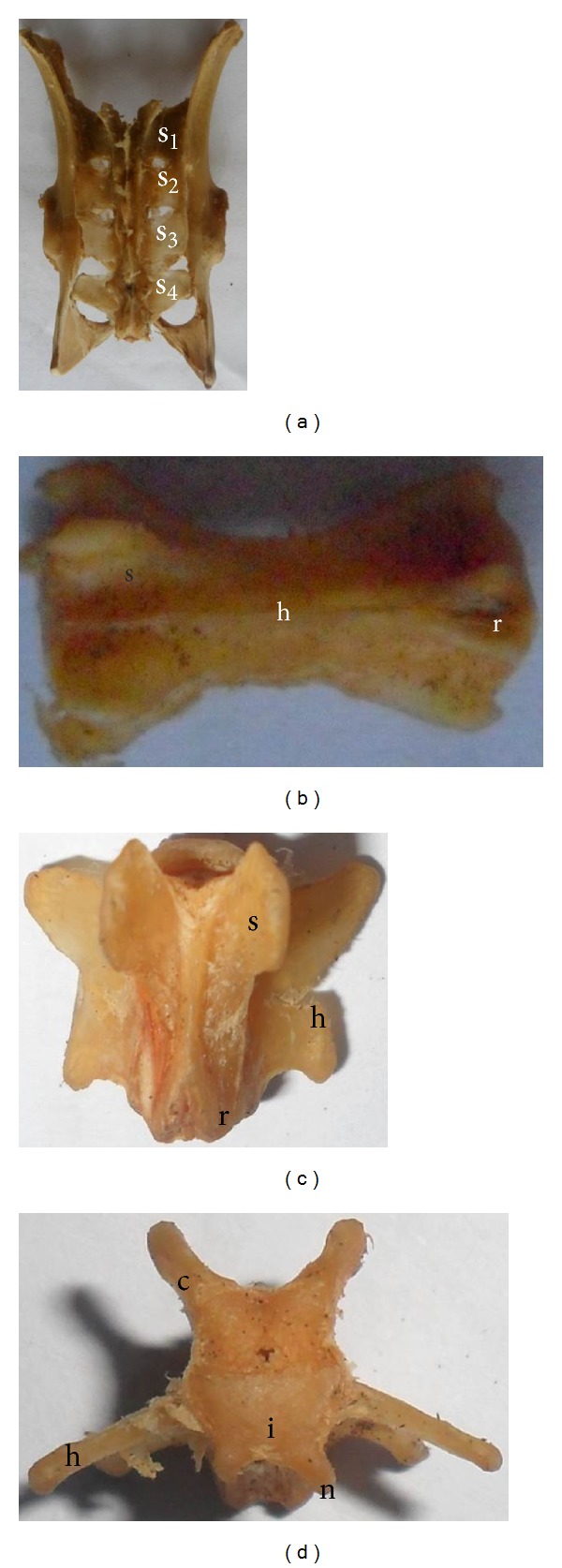
(a) dorsal view of the sacrum with the pelvic girdle; (b), (c), and (d) dorsal, cranial, and lateral views of the coccygeal vertebrae, respectively. c: cranial articular surface, h: transverse process, i: vertebral body, n: hemal arch, r: caudal articular process, and s: cranial articular process.

Os sacrum was composed of 4 sacral vertebrae, appeared fused but the outlines of the transverse and articular processes were visible in most specimens studied (Figure 3(a)). The total length of the sacrum varied from 3.8 to 4.0 cm, with individual vertebrae appearing uniform in length, while the diameter of the vertebral canal ranged between 0.2 and 0.4 cm. The fused transverse processes of S_1_ and S_2_ (pars lateralis) articulated with the ilium while the remaining two were never involved.

### 3.5. Coccygeal Vertebrae

There were 31–36 caudal or coccygeal vertebrae forming the bone of the tail with a total length ranging between 0.6 and 1.9 cm (Table 1). Cranial articular processes of the Ca_1_ to Ca_7_ caudal vertebrae were present (Figures 3(b) and 3(c)). There were rudimentary cranial articular processes from the Ca_8_ caudal vertebrae.

Ca_1, 2 & 3_ were similar to the sacrum, Ca_4, 5 & 6_, though also similarly had larger transverse processes with decreasing body lengths, spinous processes decreased in height from Ca_1_ & Ca_2_ and almost entirely diminished caudally. Hemal arch was observed at Ca_6_ and hemal processes from Ca_7_ (Figure 3(d)). Caudal parts of the coccygeal bones formed an hourglass shape, and their processes gradually became shorter; the vertebral bones also became shorter and thinner progressively towards the tip of the tail.

### 3.6. Ribs

**Figure 4:**
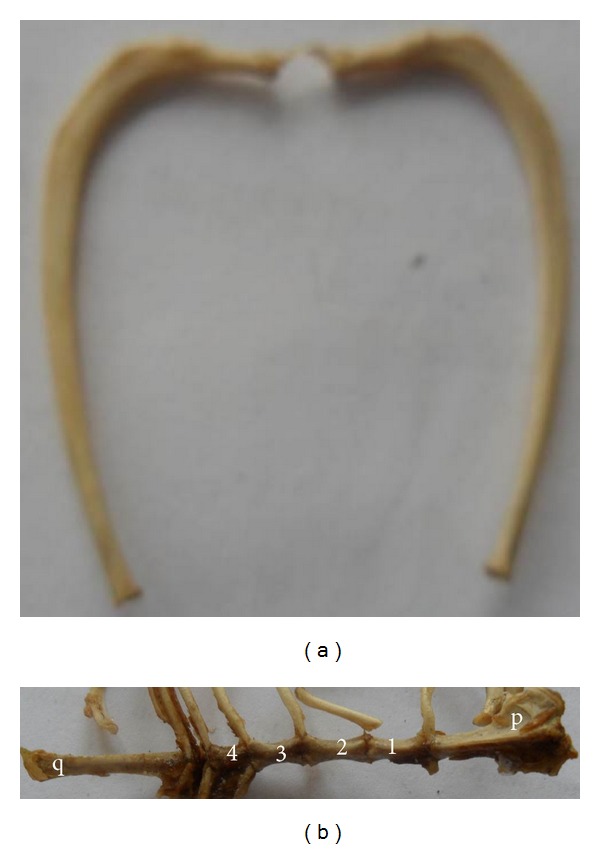
(a) lateral view of the ribs and (b) ventral view of the sternum. p: manubrium sterni, q: xiphoid process.

There were 13 pairs of ribs; the first seven pairs were sternal (costae verae) and the remaining six pairs were asternal (costae spuriae) of which the last 2-3 pairs were floating ribs (costae fluctuantes) having no distinct attachment to the costal arch. There was a distinct costal groove on the external faces of the shafts of the 3rd, 4th, and 5th ribs. The second rib had a faint groove and there were no costal grooves on the shafts of other ribs. The shafts of the first five ribs were flat, while the bodies of the other ribs were cylindrical (Figure 4(a)).

### 3.7. Sternum

The sternum was composed of deltoid-shaped manubrium sterni, four sternebrae, and slender *processus xiphoideus*. The sternebrae bodies were slender and laterally compressed. The longest sternebrae were the manubrium and xiphoid processes with average length of 1.9 cm and 2.0 cm, respectively. The second and third sternebrae were approximately 0.8 cm while the shortest was the fourth sternebra at 0.5 cm (Figure 4(b)).

## 4. Discussion

Most of the features found in this study are largely typical for rodents. The vertebral formula was reported as C_7_, T_13_, L_6_, S_4_, Ca_5_ in mole-rats [8]; C_7_, T_13_, L_6_, S_4_, Ca_6_ in the Spalacidae family [19]. In this study, it was observed to be C_7_, T_13_, L_6_, S_4_, Ca_31–36_. In the rat, the major axes of vertebral column and skull are roughly in the same horizontal line [20]. This is in consonance with results observed in mole-rats [8] as well as in this study. The vertebral column gives support to the muscles and ligaments of the back which appear firm and tough yet flexible. This probably gives the AGR great strength as it utilizes the vertebral column in motion. The homologue of cervical ribs in the Wistar rat occurs at C_6_ [15] same as in the African giant rat. All other features appeared typical for rodents. 

Mammalian sacrals are generally three to five in number [21]. In Insectivora, sacral conditions vary widely and, in moles, the vertebrae are firmly fused [22, 23]. In our study, Os sacrum was composed of 4 sacral vertebrae. The spinous processes of both sacral vertebrae and transverse processes were fused to form pars lateralis in mole-rats; as in moles [8]. In the African giant rat, the spinous processes were not fused and though the transverse processes of some had fused probably by ossification due to ageing, most were still separable only by thin connective tissue and ligaments.

In the Wistar rat, the articular processes of the coccygeal bones made no articulations caudal to Ca_3_ where the hemal arch was formed. In the AGR, the articulations continued as far as Ca_7_ bone and hemal arch was observed on Ca_6_.

In preparation of the bones, tough ligamentous attachments were observed holding the various muscles of the back and tail. The authors speculate that the strong articulations on the back and tail of the AGR form part of the explanations for the flexibility and ease with which this rodent coils up despite its sheer size and the use of the tail in the African giant rat which is reported to dig, fight, and sometimes prop itself on the tail (personal observation). 

The body of the sternum consists of five sternebrae in the mink [3], eight sternebrae in the badger [4], six sternebrae in the porcupine [6], and five sternebrae in mole-rats [8]. In this current study, four sternebrae were found in African giant rats apart from a manubrium proximally and a xiphoid process capped with a xiphoid cartilage.

In conclusion, this study demonstrates the African giant rat as a rodent with a highly flexible vertebral column as it readily coils up in defense or motion. This report presents features of the vertebral column that may be responsible for this and adds to the body of knowledge on this rodent.

## Figures and Tables

**Table 1:** Ranges of measured parameters (cm) along the vertebral column in eight adult African giant rats.

Parameters (cm)	Cervical	Thoracic	Lumbar	Sacral	Caudal
Length of vertebrae bodies	0.3–1.5	0.5–0.8	0.8–1.2	0.8–1.0	0.6–1.9
Spine length	0.2–0.6	0.4–1.2	0.6–1.2	0.5–1.0	0.2-0.3
Transverse process length	0.4–0.9	0.5–0.7	0.4-0.5	0.7–1.0	0.7
Vertebral foramen diameter	0.6–0.8	0.4–0.6	0.4–0.7	0.2–0.4	—
Vertebral foramen height	0.3–0.7	0.3-0.4	0.2–0.4	0.2-0.3	—
